# Immune Checkpoint Inhibitors: efficacy, safety, and biomarkers - a systematic review

**DOI:** 10.3389/fonc.2026.1789760

**Published:** 2026-05-20

**Authors:** Hebatalla Ismail, Anees Hassan

**Affiliations:** 1 Medical Oncology, St. Vincent’s University Hospital, UCD Cancer Centre, Dublin, Ireland; 2 Medical Oncology, University Hospital Limerick, Limerick, Ireland

**Keywords:** biomarkers, cancer immunotherapy, combination therapy, CTLA-4, immune checkpoint inhibitors, immune related adverse events, overall survival, PD-1

## Abstract

Programmed death-1 (PD-1)/programmed death ligand-1 (PD-L1) inhibitors and cytotoxic Lymphocyte-associated protein 4 (CTLA-4) inhibitors are the core classes of immune checkpoint inhibitors (ICIs) widely used in cancer treatment. These agents, including PD-1, PD-L1, and CTLA4 inhibitors, have demonstrated efficacy across multiple cancer types, enhancing progression free survival (PFS) and overall survival (OS). However, their use is associated with immune related adverse events (irAEs) that can manifest as skin rashes, hepatitis, diarrhea, colitis, hypopituitarism, and pneumonitis, among other conditions. In this systematic review, we discuss the safety and efficacy of ICIs based on 15 randomized controlled trials and cohort studies that met our eligibility criteria. All selected studies focused on analysing primary clinical outcomes including OS, PFS, objective response rate (ORR), and irAEs. These findings demonstrate that ICIs offer durable antitumor activity with favourable outcomes observed in patients with high tumour mutational burden (TMB) and PD-L1 expression. However, toxicity remains a significant concern, as some reports show substantial adverse events requiring immunosuppressive management. While PD-L1 expression and TMB are established biomarkers for predicting ICI response, their role in predicting immune-related adverse events remains investigational and is not supported by robust evidence. This review further examines combination strategies, including dual checkpoint blockade and ICI combined with chemotherapy, which have demonstrated superior clinical outcomes compared with monotherapy but are associated with increased toxicity. Future research should focus on refining patient selection criteria, optimizing toxicity management protocols, and identifying novel predictive biomarkers for ICI therapy. Understanding these aspects will facilitate the development of more effective ICI-based treatments with improved benefit-to-risk ratios, ultimately enhancing patient outcomes in oncology.

## Introduction

Immune checkpoint inhibitors (ICIs) have emerged as one of the most significant advances in cancer treatment by harnessing the immune system to target malignant cells. Conventional therapies such as chemotherapy and radiotherapy are associated with considerable toxicity and demonstrate limited efficacy in advanced-stage cancers (2). In contrast, ICIs function by blocking regulatory mechanisms that normally suppress immune activity, thereby enabling T cells to recognize and eliminate cancer cells (2). These agents primarily target CTLA-4 and the PD-1/PD-L1 axis, which are critical immune checkpoint pathways exploited by cancer cells to evade immune surveillance (3).

Ipilimumab, a monoclonal antibody targeting CTLA-4, was the first ICI to demonstrate clinical efficacy in metastatic melanoma (4). Subsequently, PD-1 inhibitors including nivolumab and pembrolizumab, as well as PD-L1 inhibitors such as atezolizumab and durvalumab, have been approved for multiple indications including non-small cell lung cancer (NSCLC), renal cell carcinoma, and Hodgkin lymphoma (5–7). Unlike conventional treatments, ICIs have demonstrated durable responses and the potential for long-term survival in select patient populations, with benefits persisting even after treatment discontinuation (8). Despite these advances, response rates vary considerably among patients, highlighting the need for predictive biomarkers to guide treatment selection (9).

ICIs are associated with a unique toxicity profile characterized by immune-related adverse events (irAEs) resulting from immune system activation against normal tissues. These toxicities can affect multiple organ systems, with the most common manifestations including cutaneous reactions, diarrhea, colitis, endocrine dysfunction, hepatitis, and pneumonitis (10). Rare but serious complications such as myocarditis and neurological toxicities require prompt recognition and aggressive management (11). The mechanisms underlying irAE development remain incompletely understood; however, current evidence suggests that checkpoint blockade disrupts immune tolerance, leading to autoimmune-like tissue injury (12). Corticosteroids and other immunosuppressive agents represent the mainstay of irAE management, though optimal treatment algorithms continue to evolve (13).

Combination immunotherapy strategies, in which ICIs are combined with other immune modulators or conventional therapies, have demonstrated improved efficacy in certain settings. For example, the combination of nivolumab and ipilimumab has shown superior outcomes in melanoma and renal cell carcinoma, albeit with increased toxicity (14, 55). Ongoing investigations are exploring novel checkpoint targets such as lymphocyte-activation gene 3 (LAG-3) and T-cell immunoglobulin and mucin-domain containing-3 (TIM-3) to enhance current immunotherapeutic approaches.

Given the complexity of immune checkpoint blockade, the future of ICI therapy lies in personalized treatment approaches integrating biomarkers such as PD-L1 expression, TMB, and gut microbiome composition to predict response and toxicity (9). Emerging technologies including artificial intelligence and machine learning may facilitate patient stratification and treatment optimization based on individual risk-benefit profiles (15).

As the use of ICIs in cancer treatment continues to expand, a comprehensive understanding of their efficacy and safety profile is essential to maximize therapeutic benefit while minimizing adverse events. This review critically examines the current evidence regarding ICI efficacy, toxicity profiles, predictive biomarkers, and future directions to advance the field of cancer immunotherapy.

## Materials and methods study design

This systematic review adheres to the Preferred Reporting Items for Systematic Reviews and Meta-Analyses (PRISMA) guidelines to ensure a rigorous and methodologically sound assessment of the safety and efficacy of immune checkpoint inhibitors (ICIs). The systematic review targeted articles from 2010 to 2024, including peer-reviewed clinical trials and observational studies. Each study on the use of ICIs was reviewed systematically to delineate the mechanisms of action, primary efficacy outcomes, and immune-related adverse events (irAEs) that have been reported. The study incorporated both qualitative and quantitative approaches to analyze the available literature, focusing on trends, patterns, and research gaps in clinical ICI use while working toward developing evidence-based recommendations. This systematic review was not prospectively registered in PROSPERO as data extraction had commenced prior to registration. The methodology adhered to PRISMA guidelines to ensure transparency and reproducibility.

### Selection criteria

While compiling the current review, studies were selected according to criteria of relevance and methodological quality. The review prioritized clinical trials and high-quality observational studies across different types of malignancies. Systematic reviews and meta-analyses were reviewed during the background literature search to inform the scope of the review and identify relevant primary studies; however, only primary research studies were included in the final synthesis to avoid duplication of data and ensure methodological consistency. Preferably, prospective and retrospective approaches were used in the identified investigations, with small sample-size studies, non-peer-reviewed publications, and articles reporting preliminary findings excluded from this review.

Given the large volume of literature on immune checkpoint inhibitors, a rigorous prioritization framework was applied to ensure inclusion of only the most methodologically robust and clinically informative studies. Studies were prioritized based on the following criteria: (1) sample size sufficient to detect clinically meaningful differences in primary outcomes (minimum 100 participants for RCTs, 200 for cohort studies); (2) adequate follow-up duration to capture survival endpoints (minimum 12 months); (3) comprehensive reporting of both efficacy and safety outcomes; and (4) low to moderate risk of bias as assessed by standardized evaluation tools. This stringent approach accounts for the reduction from 4,216 initially identified records to 15 included studies and was implemented to ensure that the conclusions of this review are based on high-quality evidence.

### Inclusion criteria

Studies were included if they met the following criteria: (1) peer-reviewed randomized controlled trials (RCTs), cohort studies, or case-control studies evaluating the efficacy and safety of ICIs; (2) publications in English from 2010 to 2024; (3) studies focusing on FDA-approved ICIs such as pembrolizumab, nivolumab, atezolizumab, durvalumab, and ipilimumab; (4) studies that reported clinical outcomes, including survival rates, response rates, and immune-related adverse events; (5) research analysing predictive biomarkers for ICI efficacy and toxicity; and (6) real-world evidence studies assessing long-term benefits and toxicity management strategies in patients receiving ICIs.

### Exclusion criteria

Studies were excluded if they met the following criteria: (1) systematic reviews, meta-analyses, case reports, case series, conference abstracts, and expert opinions that did not provide primary data; (2) non-English publications due to feasibility constraints; (3) studies focusing on preclinical models, *in vitro* experiments, or animal studies that did not include human subjects; (4) trials investigating combination therapies without clear stratification of ICI effects; (5) articles with incomplete or insufficient data on safety and efficacy outcomes; and (6) studies with a high risk of bias, as assessed by standardized evaluation tools.

### Search strategy

A comprehensive literature search was conducted in October 2024 using the following electronic databases: PubMed (via MEDLINE), Embase (via OvidSP), Scopus, Web of Science Core Collection, and the Cochrane Central Register of Controlled Trials (CENTRAL). The search strategy combined controlled vocabulary terms (Medical Subject Headings [MeSH] in PubMed; Emtree terms in Embase) with free-text keywords to maximize sensitivity.

The search incorporated terms related to immune checkpoint inhibitors (including “immune checkpoint inhibitor,” “PD-1,” “PD-L1,” “CTLA-4,” and individual drug names such as ipilimumab, nivolumab, pembrolizumab, atezolizumab, durvalumab, avelumab, cemiplimab, dostarlimab, toripalimab, and tislelizumab), combined with terms for malignancy (including “neoplasm,” “cancer,” “carcinoma,” “tumor,” and “malignancy”) and study design filters for clinical trials. Boolean operators (AND, OR) were used to combine search concepts. The complete search strategies for all databases, including exact search strings, are provided in **Supplementary Table 1**.

The database searches yielded a total of 4,216 records: PubMed (n=1,180), Embase (n=1,350), Scopus (n=820), Web of Science (n=650), and Cochrane CENTRAL (n=216). Additional searches were conducted in ClinicalTrials.gov to identify completed trials with published results. Grey literature sources, including regulatory documents from the U.S. Food and Drug Administration (FDA) and the European Medicines Agency (EMA), were reviewed to supplement the primary search. Reference lists of all included studies and relevant systematic reviews identified during the search process were manually screened to identify additional eligible studies not captured by the electronic database searches.

### Study selection process

**Figure 1:**
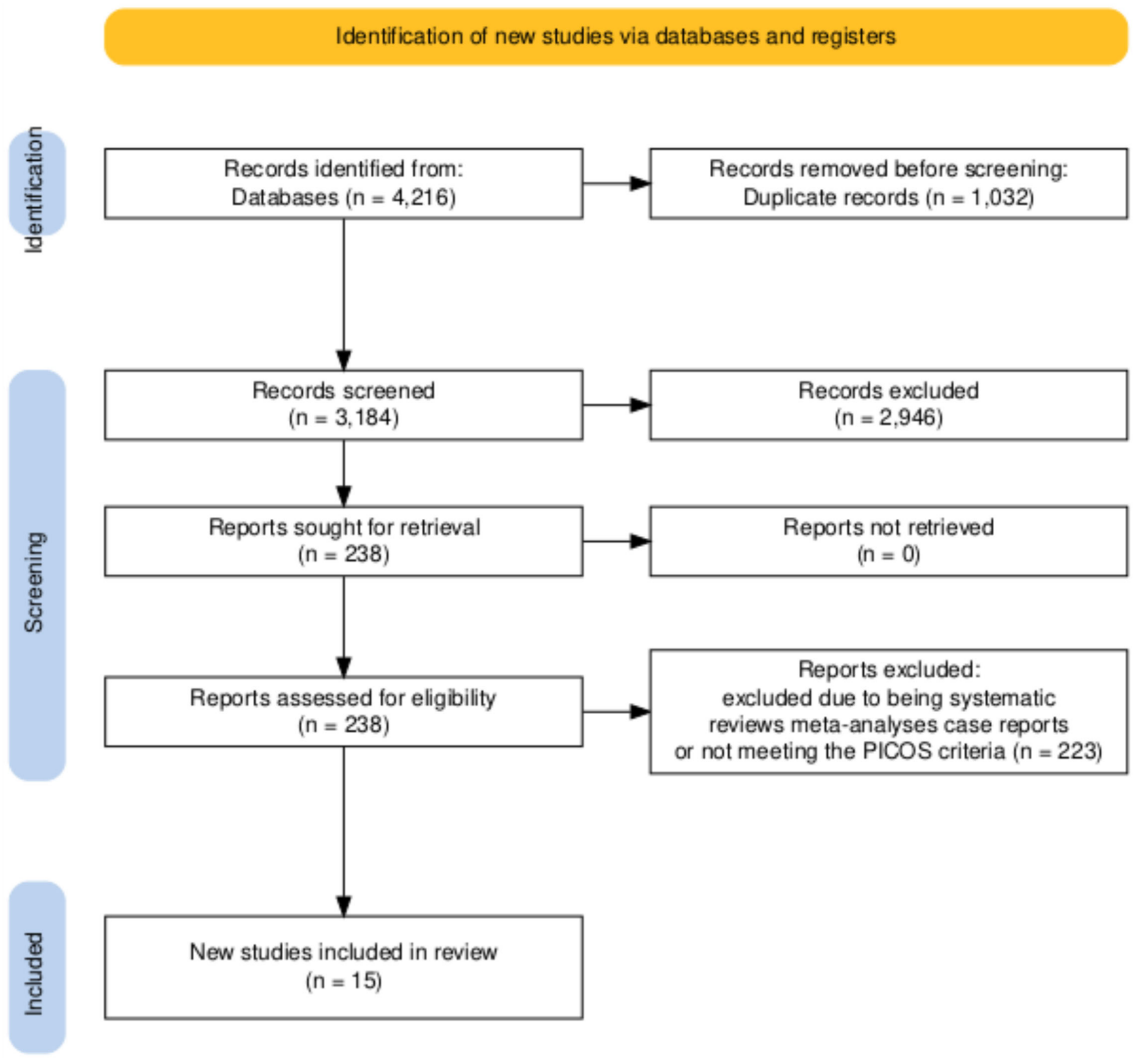
PRISMA flowchart.

Following the removal of 1,032 duplicates, 3,184 records remained for title and abstract screening. Two reviewers (HI, AH) independently screened all titles and abstracts against the predefined eligibility criteria. Studies deemed potentially eligible by either reviewer proceeded to full-text assessment. Of these, 2,946 records were excluded based on title and abstract review due to irrelevance, non-human studies, or lack of efficacy and safety data on immune checkpoint inhibitors. The full texts of 238 studies were retrieved for detailed evaluation. Following eligibility assessment, 223 studies were excluded for the following reasons: systematic reviews and meta analyses (n=87), case reports and case series (n=62), insufficient outcome data (n=41), and failure to meet PICOS criteria (n=33). Disagreements between reviewers were resolved through discussion until consensus was reached. Ultimately, 15 studies were included in this systematic review. The study selection process is illustrated in the PRISMA flow diagram (**Figure 1**).

### Study question

The primary research question guiding this systematic review was: “What are the safety and efficacy profiles of immune checkpoint inhibitors in cancer therapy, and how do immune related adverse events impact clinical outcomes?” This question was developed based on the PICOS (Population, Intervention, Comparison, Outcome, and Study design) framework to ensure a structured approach to data extraction and synthesis. The specific components of this framework are summarized in **Table 1**.

**Table 1:** PICOS framework for research question.

Component	Description
Population	Patients with various malignancies receiving immune checkpoint inhibitors
Intervention	Administration of PD-1, PD-L1, or CTLA-4 inhibitors (e.g., pembrolizumab, nivolumab, ipilimumab)
Comparison	Standard chemotherapy, targeted therapy, or placebo where applicable
Outcome	Efficacy (overall survival, progression-free survival) and safety (immunerelated adverse events)
Study Design	Randomized controlled trials, cohort studies, and case-control studies

### Data extraction

Data were extracted by two investigators using a standardized extraction form. The extracted data points included study details (author, year, country, study design, and sample size), patient characteristics (age, gender, cancer type), interventions (type and dosage of ICI used), primary outcomes (objective response rate, progression-free survival, overall survival), and safety endpoints (incidence and severity of irAEs). To minimize discrepancies in data extraction, the results were reviewed by a third researcher to enhance inter-observer reliability.

### Study outcomes

The primary endpoints in the present analysis were the effectiveness of ICIs in terms of PFS, OS, and response rates across various cancers. Regarding safety outcomes, the incidence, severity, and management of irAEs according to organ class were examined. Secondary objectives included defining the utility of biomarkers to distinguish responders from non-responders and to predict toxicity risk, as well as evaluating strategies for optimizing the therapeutic window of ICIs.

#### Risk of bias assessment of individual studies

Two reviewers (HI, AH) independently assessed the risk of bias for each included study. Randomized controlled trials were evaluated using the Cochrane Risk of Bias 2 (RoB 2) tool across five domains: randomization process, deviations from intended interventions, missing outcome data, measurement of the outcome, and selection of the reported result. Each domain was rated as “low risk,” “some concerns,” or “high risk,” and an overall judgment was assigned accordingly. Observational studies were assessed using the Newcastle-Ottawa Scale (NOS), which assigns up to 9 stars across three domains: selection (maximum 4 stars), comparability (maximum 2 stars), and outcome assessment (maximum 3 stars). Studies scoring 7–9 stars were classified as high quality, 4–6 as moderate quality, and 0–3 as low quality. Disagreements between reviewers were resolved through discussion or consultation with a third reviewer. To minimize the introduction of bias, only studies classified as having low or moderate risk of bias were included in the synthesis. Individual study level risk of bias ratings are presented in **Supplementary Table SX**.

#### Analytical approach

This review was designed as a qualitative systematic review rather than a quantitative metanalysis. Although some outcome data appeared numerically comparable across studies, substantial clinical and methodological heterogeneity precluded meaningful statistical pooling. Sources of heterogeneity included: (1) variation in tumour types and disease stages across studies; (2) differences in comparator arms (placebo, standard chemotherapy, or targeted therapy); (3) inconsistent definitions and reporting of immune-related adverse events; (4) variable follow-up durations; and (5) differences in patient populations, including prior treatment exposure and performance status. Given these limitations, a narrative synthesis was deemed the most appropriate approach to accurately represent the evidence while avoiding potentially misleading pooled estimates. This methodology aligns with Cochrane guidance, which recommends against meta-analysis when substantial heterogeneity exists that cannot be adequately explained (44). To maintain methodological transparency, the Discussion section clearly distinguishes findings derived directly from the 15 included primary studies from contextual evidence drawn from the broader literature. The latter is referenced to provide clinical interpretation and mechanistic context but is explicitly identified as external to the formal evidence synthesis.

#### Certainty of evidence assessment

The certainty of the body of evidence for each primary outcome (overall survival, progression-free survival, objective response rate, and incidence of immune-related adverse events) was assessed using the Grading of Recommendations Assessment, Development and Evaluation (GRADE) framework. Two reviewers independently rated the certainty of evidence as high, moderate, low, or very low based on five domains: risk of bias (informed by the RoB 2 and NOS assessments described above), inconsistency across studies, indirectness of evidence, imprecision of effect estimates, and publication bias. GRADE was applied at the outcome level across the body of evidence, not as a study-level quality tool. Publication bias was assessed qualitatively, as the absence of meta analysis precluded the use of funnel plots or Egger’s test. GRADE assessments for each outcome are summarized in a Summary of Findings table (**Supplementary Table SY**).

### Results study selection

As described in the Methods section, the systematic search and screening process yielded 15 studies for inclusion. The study selection process is illustrated in the PRISMA flow diagram (**Figure 1**). **Table 2** summarizes the characteristics of the included studies, detailing the interventions, sample sizes, and key outcomes for each study. **Table 3** presents the risk of bias assessment for each of the included studies. **Table 4** provides a summary of key efficacy and safety outcomes across included studies organized by tumor type. The GRADE certainty of evidence ratings for each primary outcome are presented in **Supplementary Table SY**. The certainty of evidence was rated as moderate for overall survival outcomes in melanoma and NSCLC, where multiple RCTs with consistent findings were available, and low for safety outcomes across all tumour types due to inconsistent irAE definitions, variable grading criteria, and imprecision in event rates.

**Table 2:** Characteristics of the included studies.

Author, Year	Country	Study design	Sample size	Patient demographics	Interventi on (ICI used)	Primary efficacy outcomes	Safety outcomes (irAEs)	Outcomes	Findings
Hodi et al., 2010 (4)	USA	RCT	676	Advanced melanoma	Ipilimuma b	OS signific antly improv ed	High incidence of colitis	OS: 10.1 months vs. 6.4 months	Ipilimumab improved survival but had high toxicity
Garon et al., 2015 (5)	USA	RCT	305	NSCLC	Pembroliz umab	PFS and OS benefit	Fatigue, pneumoni tis	OS: 12.2 months vs. 9.4 months	Pembrolizumab outperformed chemotherapy in PD-L1 positive NSCLC
Motzer et al., 2015 (6)	USA	RCT	821	Renal Cell Carcinoma	Nivoluma b	PFS improv ed	Fatigue, immune- related toxicities	OS: 25 months vs. 19.6 months	Nivolumab showed superior survival over everolimus
Ansell et al., 2015 (7)	USA	Cohort Study	95	Hodgkin’s Lymphoma	Nivoluma b	High ORR	Mild irAEs	ORR: 87%	Nivolumab demonstrated a high response rate in relapsed Hodgkin’s lymphoma
Wolcho k et al., 2017 (14)	USA	RCT	945	Advanced melanoma	Nivoluma b + Ipilimuma b	OS signific antly improv ed	Severe irAEs reported	OS: 58% at 3 years	Combination therapy improved survival but increased toxicity
Reck et al., 2016 (16)	Multi ple	RCT	305	NSCLC	Pembroliz umab	PFS and OS benefit	Fatigue, diarrhea	OS: 13.7 months vs. 10.3 months	Pembrolizumab was superior to chemotherapy in PD-L1 positive patients
Ferris et al., 2016 (17)	USA	RCT	361	Squamous Cell Carcinoma	Nivoluma b	OS improv ed	Pneumoni tis, fatigue	OS: 7.5 months vs. 5.1 months	Nivolumab improved survival in recurrent head and neck SCC
Bellmun t et al., 2017 (18)	USA	RCT	542	Urothelial Carcinoma	Pembroliz umab	PFS improv ed	Colitis, rash	OS: 10.3 months vs. 7.4 months	Pembrolizumab was superior to chemotherapy as second-line therapy
Ribas et al., 2016 (19)	USA	Cohort Study	655	Advanced melanoma	Pembroliz umab	Tumor respons e correlat ed with survival	Skin rash, fatigue	ORR: 34%	Tumor response was predictive of long-term survival
Herbst et al., 2016 (20)	Multi ple	RCT	1034	NSCLC	Pembroliz umab	PFS and OS benefit	Immune- related toxicities	OS: 13.5 months vs. 8.2 months	Pembrolizumab showed better survival than docetaxel in PDL1 positive patients
Larkin et al., 2015 (21)	USA	RCT	945	Advanced melanoma	Nivoluma b + Ipilimuma b	OS improv ed	Fatigue, colitis	OS: 11.5 months vs. 6.9 months	Combination therapy outperformed monotherapy but had higher toxicity
Borghae i et al., 2015 (22)	USA	RCT	582	NSCLC	Nivoluma b	OS benefit vs. docetax el	Diarrhea, hepatitis	OS: 12.2 months vs. 9.6 months	Nivolumab improved OS in advanced NSCLC
Eggerm ont et al., 2018 (23)	Multi ple	RCT	1019	Melanoma Stage III	Pembroliz umab	DFS improv ed	Fatigue, dermatiti s	DFS: 75.4% vs. 61%	Adjuvant pembrolizumab significantly improved disease-free survival
Motzer et al., 2018 (55)	USA	RCT	1096	Renal Cell Carcinoma	Nivoluma b + Ipilimuma b	OS signific antly improv ed	Diarrhea, hepatitis	OS: 75% vs. 60% at 18 months	Combination therapy showed superior OS over sunitinib
Postow et al., 2015 (1)	USA	RCT	945	Advanced melanoma	Nivoluma b + Ipilimuma b	OS signific antly improv ed	Colitis, pneumoni tis	OS: 74% vs. 53% at 12 months	Combination therapy significantly improved survival in melanoma

**Table 3:** Risk of bias assessment.

Author, Year	Study design	Randomization bias	Allocation concealment bias	Blinding bias	Incomplete outcome data bias	Selective reporting bias	Overall risk of bias
Hodi et al., 2010 (4)	RCT	Low	Low	Modera te	Low	Low	Low
Garon et al., 2015 (5)	RCT	Low	Low	Low	Low	Low	Low
Motzer et al., 2015 (6)	RCT	Low	Low	Low	Low	Low	Low
Ansell et al., 2015 (7)	Cohor t Study	Moderate	Moderate	High	Moderate	Moderat e	Modera te
Wolchok et al., 2017 (14)	RCT	Low	Low	Low	Low	Low	Low
Reck et al., 2016 (16)	RCT	Low	Low	Low	Low	Low	Low
Ferris et al., 2016 (17)	RCT	Low	Low	Low	Low	Low	Low
Bellmunt et al., 2017 (18)	RCT	Low	Low	Low	Low	Low	Low
Ribas et al., 2016 (19)	Cohor t Study	Moderate	Moderate	High	Moderate	Moderat e	Modera te
Herbst et al., 2016 (20)	RCT	Low	Low	Low	Low	Low	Low
Larkin et al., 2015 (21)	RCT	Low	Low	Low	Low	Low	Low
Borghaei et al., 2015 (22)	RCT	Low	Low	Low	Low	Low	Low
Eggermo nt et al., 2018 (23)	RCT	Low	Low	Low	Low	Low	Low
Motzer et al., 2018 (6)	RCT	Low	Low	Low	Low	Low	Low
Postow et al., 2015 (1)	RCT	Low	Low	Low	Low	Low	Low

**Table 4:** Summary of key efficacy and safety outcomes across included studies by tumor type.

Tumor type	No. of studies	ICI regimen(s)	Key efficacy outcomes	Grade 3–4 irAE rate	Biomarker reported	References
Advanced Melanoma	5	Ipi; Nivo+Ipi; Pembro	OS 10.1 mo (Ipi); 58% 3-yr OS (Nivo+Ipi); ORR 34% (Pembro)	16–59%	PD-L1 expression	(1, 4, 14, 19, 21)
NSCLC	4	Pembro; Nivo	OS 12.2–13.7 mo vs 8.2–10.3 mo (chemo)	10–15%	PD-L1 ≥50%	(5, 16, 20, 22)
Renal Cell Carcinoma	2	Nivo; Nivo+Ipi	OS 25 mo vs 19.6 mo (everolimus); 75% vs 60% 18-mo OS	19–46%	PD-L1 expression	(6, 55)
Head and Neck SCC	1	Nivo	OS 7.5 mo vs 5.1 mo (standard)	13%	Not reported	(17)
Urothelial Carcinoma	1	Pembro	OS 10.3 mo vs 7.4 mo (chemo)	15%	PD-L1 expression	(18)
Hodgkin Lymphoma	1	Nivo	ORR 87%	Low (mild irAEs)	Not reported	(7)
Stage III Melanoma (adjuvant)	1	Pembro	DFS 75.4% vs 61% (placebo)	14%	Not reported	(23)

Ipi, ipilimumab; Nivo, nivolumab; Pembro, pembrolizumab; OS, overall survival; ORR, objective response rate; DFS, disease-free survival; irAE, immune-related adverse event; NSCLC, non-small cell lung cancer; SCC, squamous cell carcinoma.

## Discussion

The following discussion synthesizes findings from the 15 included primary studies and contextualizes these results within the broader literature on immune checkpoint inhibitors. Where appropriate, additional published evidence, including clinical practice guidelines, mechanistic studies, and real-world data, has been referenced to provide comprehensive interpretation of the findings and their clinical implications. This approach ensures that the critical analysis extends beyond the scope of the included studies while maintaining transparency regarding the sources of evidence.

Immune checkpoint inhibitors (ICIs) have revolutionized cancer treatment by enhancing antitumor immune surveillance. These agents, including PD-1, PD-L1, and CTLA-4 inhibitors, have demonstrated efficacy across multiple tumor types and have been shown to improve PFS and OS across various stages of cancer (24). Nevertheless, their clinical efficacy is accompanied by the development of immune-related adverse events affecting multiple organ systems, which may necessitate modifications to the patient’s treatment plan (1). Balancing efficacy and safety remains central to the effective use of ICIs in cancer treatment. However, the heterogeneity of patient populations, tumour biology, and study designs across trials presents significant challenges in generalizing these findings to routine clinical practice (35).

### Synthesis of efficacy findings from included studies

Among the 15 studies included in this systematic review, 13 were randomized controlled trials and 2 were cohort studies evaluating ICI efficacy across six tumour types. Overall survival improvements were consistently demonstrated across most tumour types and treatment settings.

In advanced melanoma, five included studies evaluated ICIs. Hodi et al. (4) demonstrated that ipilimumab improved OS to 10.1 months compared with 6.4 months with gp100 vaccine alone (4). Combination nivolumab plus ipilimumab achieved superior outcomes, with Wolchok et al. (14) reporting 3-year OS of 58% (14), Larkin et al. (21) reporting OS of 11.5 months versus 6.9 months with monotherapy (21), and Postow et al. (1) demonstrating 12-month OS of 74% versus 53% (1). Ribas et al. (19) showed that tumour response to pembrolizumab was predictive of long-term survival, with an ORR of 34% (19). In the adjuvant setting, Eggermont et al. (23) demonstrated that pembrolizumab significantly improved disease-free survival (75.4% vs 61%) in resected stage III melanoma (23).

In NSCLC, four included RCTs demonstrated consistent PD-1 inhibitor efficacy in PD-L1-positive patients. Garon et al. (5) reported OS of 12.2 months versus 9.4 months with chemotherapy (5). Reck et al. (16) demonstrated OS of 13.7 months versus 10.3 months (16), and Herbst et al. (20) showed OS of 13.5 months versus 8.2 months with docetaxel (20). Borghaei et al. (22) reported nivolumab OS of 12.2 months versus 9.6 months with docetaxel in nonsquamous NSCLC (22).

In renal cell carcinoma, Motzer et al. (6) demonstrated nivolumab superiority over everolimus with OS of 25 months versus 19.6 months (6). The same group later showed that combination nivolumab plus ipilimumab achieved 18-month OS of 75% versus 60% with sunitinib (55). In head and neck squamous cell carcinoma, Ferris et al. (17) reported nivolumab OS of 7.5 months versus 5.1 months with standard therapy (17). In urothelial carcinoma, Bellmunt et al. (18) demonstrated pembrolizumab OS of 10.3 months versus 7.4 months with chemotherapy (18). Finally, in relapsed Hodgkin lymphoma, Ansell et al. (7) reported an ORR of 87% with nivolumab (7).

#### Contextualization with broader literature

These findings from the included studies are consistent with the broader ICI literature. The CheckMate 227 trial, which was published after our search period and therefore not included in this review, similarly demonstrated that nivolumab plus ipilimumab was superior to chemotherapy in NSCLC (25). Long-term follow-up data from KEYNOTE-001, also beyond our inclusion timeframe, have shown durable responses exceeding five years with pembrolizumab in advanced melanoma (26). Collectively, the evidence supports substantial ICI effectiveness, particularly in tumours with high immunogenicity (27).

Despite the consistent survival benefits observed across the 15 included studies, several limitations of this evidence base warrant consideration. First, the majority of pivotal trials enrolled highly selected patient populations with good performance status and limited comorbidities, which may not reflect the broader oncology population encountered in clinical practice (36). Second, considerable heterogeneity exists in efficacy outcomes across different tumor types and histological subtypes, suggesting that the benefits of ICIs are not uniform. Notably, the included studies were concentrated in melanoma (5 studies), NSCLC (4 studies), and renal cell carcinoma (2 studies), with only single studies in head and neck SCC, urothelial carcinoma, and Hodgkin lymphoma. This limits the generalizability of findings to tumour types not represented in the included evidence base, such as pancreatic and prostate cancer, where ICI efficacy remains limited (27, 37). Third, the definition and assessment of clinical endpoints varied across studies, with some trials using investigator-assessed progression-free survival rather than independent central review, potentially introducing measurement bias (38). These factors collectively limit the direct comparability of results across trials and underscore the importance of interpreting efficacy data within the context of individual study designs.

### Safety and immune-related adverse events

All 15 included studies reported immune-related adverse events, with toxicity profiles varying by ICI class and treatment regimen. The most frequently reported irAEs across studies were fatigue, skin reactions (rash, dermatitis), gastrointestinal toxicities (diarrhea, colitis), and endocrine dysfunction.

Combination nivolumab plus ipilimumab was consistently associated with higher toxicity rates compared with monotherapy. Wolchok et al. (14) reported severe irAEs with combination therapy in advanced melanoma (14). Larkin et al. (21) similarly documented fatigue and colitis as predominant toxicities with combination treatment (21), and Postow et al. (1) reported colitis and pneumonitis as significant adverse events (1). Motzer et al. (55) observed diarrhea and hepatitis with combination therapy in renal cell carcinoma (55).

Among PD-1 inhibitor monotherapy studies, the toxicity profile was generally more favourable. In NSCLC, Garon et al. (5) reported fatigue and pneumonitis as the most common irAEs with pembrolizumab (5). Reck et al. (16) documented fatigue and diarrhoea (16), while Herbst et al. (20) and Borghaei et al. (22) reported immune-related toxicities including diarrhoea and hepatitis (20, 22). In head and neck SCC, Ferris et al. (17) observed pneumonitis and fatigue with nivolumab (17). Bellmunt et al. (18) reported colitis and rash with pembrolizumab in urothelial carcinoma (18).

CTLA-4 inhibitor monotherapy demonstrated the highest rates of gastrointestinal toxicity. Hodi et al. (4) reported a high incidence of colitis with ipilimumab in melanoma (4). In contrast, Ansell et al. (7) reported only mild irAEs with nivolumab monotherapy in Hodgkin lymphoma (7), and the cohort studies by Ribas et al. (19) documented skin rash and fatigue as predominant toxicities with pembrolizumab (19). Eggermont et al. (23) reported fatigue and dermatitis in the adjuvant pembrolizumab setting (23).

#### Contextualization with broader literature

The safety findings from the included studies align with the broader irAE literature. These toxicities occur when checkpoint blockade leads to immune system overactivation, resulting in inflammation of normal tissues (10). Commonly affected organ systems include the skin, gastrointestinal tract, endocrine glands, liver, and respiratory system. Thyroiditis, adrenal insufficiency, and hypophysitis are frequently associated with CTLA-4 inhibitors, whereas pneumonitis is more common with PD-1 inhibitors (13). Severe irAEs such as myocarditis and neurotoxicity, although rare, can be fatal if not detected and managed promptly (11).

Among the 15 included studies, the specific grading criteria and severity thresholds for adverse event reporting varied considerably, with some trials using different versions of the Common Terminology Criteria for Adverse Events (CTCAE), limiting direct cross-study comparisons (39). Furthermore, the incidence of irAEs may be underestimated in clinical trials due to stringent eligibility criteria that exclude patients with pre-existing autoimmune conditions or organ dysfunction, who may be at higher risk for immune-mediated toxicities (40). Real-world evidence suggests that irAE rates in routine clinical practice may exceed those reported in controlled trials, emphasizing the need for vigilant monitoring in all patients receiving ICIs (10, 41). Additionally, the long-term sequelae of irAEs, particularly endocrinopathies requiring lifelong hormone replacement, represent an underappreciated burden that warrants greater attention in both clinical trials and practice guidelines (42).

### Management of irAEs

#### Implications of included studies for irAE management

Although the 15 included studies were not designed to evaluate irAE management strategies, the safety data reported provide important context for clinical practice. The high incidence of colitis observed with ipilimumab in Hodi et al. (2010, 4) and with combination therapy in Postow et al. (2015, 1), Larkin et al. (2015, 21), and Wolchok et al. (2017, 14) underscores the importance of early gastrointestinal toxicity recognition. Similarly, the pneumonitis reported with PD-1 inhibitors in Garon et al. (2015, 5) and Ferris et al. (2016, 17) highlights the need for respiratory monitoring. The generally mild irAE profile observed with nivolumab monotherapy in Hodgkin lymphoma by Ansell et al. (2015, 7) suggests that toxicity burden varies substantially by disease context and regimen intensity. These findings from the included studies reinforce the need for risk-stratified monitoring approaches.

#### Contextualization with broader literature

As with other immune-related adverse events, the management of irAEs requires a multidisciplinary team approach, with early identification being critical to reducing morbidity (10). For moderate to severe irAEs, corticosteroids are recommended in accordance with ASCO and ESMO guidelines, while infliximab or mycophenolate mofetil may be considered in steroid-refractory cases (28). Additionally, recent investigations suggest that the gut microbiome may influence both ICI efficacy and toxicity through modulation of immune activity (43). This has created opportunities for microbiome-based treatment approaches to mitigate irAE toxicity while preserving antitumor activity.

However, current management guidelines are largely based on expert consensus and retrospective data rather than prospective randomized trials, representing a significant evidence gap (33). The optimal duration of immunosuppressive therapy, the impact of corticosteroids on antitumor efficacy, and the long-term outcomes of patients who experience severe irAEs remain inadequately characterized (45). Emerging data suggest that patients who develop certain irAEs may paradoxically experience improved tumor responses, raising important questions about the relationship between immune activation, toxicity, and efficacy that require further investigation (28, 46). Clinicians must therefore navigate considerable uncertainty when balancing the risks of undertreating potentially serious irAEs against the potential for compromising antitumor immunity with aggressive immunosuppression.

Importantly, while PD-L1 expression and TMB have demonstrated value as predictive biomarkers for ICI response, the evidence supporting their use as predictors of immune-related adverse event risk is substantially weaker. A meta-regression analysis by Osipov et al. (56) found that TMB was significantly associated with objective response rate (P < 0.0001) but was not significantly associated with toxicity across ICI treatment groups (56). Current guidelines from the College of American Pathologists endorse PD-L1 and TMB testing for treatment selection based on response prediction, not for toxicity risk stratification (57).

These findings underscore the need for distinct biomarker strategies for efficacy prediction versus toxicity prediction, as the biological mechanisms underlying antitumour immunity and autoimmune toxicity may not be fully overlapping. Wang et al. (58) conducted a real-world cohort study characterizing immune-related adverse events in patients receiving immune checkpoint inhibitors, demonstrating the complexity of irAE patterns across treatment regimens and the importance of real-world data in complementing clinical trial safety profiles (58), while Wijnen et al. (59) highlighted that advances in single-cell and spatial transcriptomics now enable high-resolution analysis of tumour–immune interactions that drive both therapy sensitivity and immune-related adverse events, supporting the integration of computational multi-biomarker models for personalized immunotherapy (59). Together, these studies reinforce the need for integrated, multi-biomarker approaches that separately address response prediction and toxicity risk assessment.

### Biomarker evidence from included studies

Among the 15 included studies, PD-L1 expression was the most frequently evaluated predictive biomarker, particularly in NSCLC trials. Garon et al. (5) demonstrated that pembrolizumab efficacy correlated with PD-L1 expression levels, with superior outcomes observed in patients with PD-L1 tumour proportion scores ≥50% (5). Reck et al. (16) confirmed that pembrolizumab was superior to chemotherapy specifically in PD-L1-positive NSCLC patients (16), and Herbst et al. (20) similarly showed improved survival with pembrolizumab over docetaxel in PD-L1-positive patients (20). These findings established PD-L1 expression as a clinically actionable biomarker for patient selection in NSCLC.

In contrast, the melanoma and renal cell carcinoma studies included in this review did not use PD-L1 expression as a primary selection criterion, yet demonstrated substantial ICI efficacy regardless of PD-L1 status. Hodi et al. (2010, 4), Wolchok et al. (2017, 14), Larkin et al. (2015, 21), and Motzer et al. (2015, 2018, 6, 55) all reported survival benefits without PD-L1-based patient selection, suggesting that the predictive value of PD-L1 varies by tumour type. Ribas et al. (19) demonstrated that tumour response itself was predictive of long-term survival in melanoma, independent of baseline biomarker status (19).

None of the 15 included studies prospectively evaluated tumour mutational burden (TMB) as a selection biomarker, reflecting the timeframe of the included trials (2010–2018) before TMB assessment became widely available.

#### Contextualization with broader literature

The biomarker findings from the included studies align with the broader literature while highlighting important limitations. PD-L1 expression remains the most widely used biomarker for PD-1/PD-L1 inhibitor therapy; however, its predictive value is highly context-dependent and varies considerably according to tumour histology, assay methodology, and treatment setting (29). A significant limitation is the lack of standardization across assays. Multiple immunohistochemistry platforms exist, including 22C3, 28-8, SP142, and SP263, each with different antibodies, scoring systems, and positivity thresholds, leading to substantial inter-assay variability and challenges in cross-trial comparisons (46). Furthermore, PD-L1 expression demonstrates spatial and temporal heterogeneity, meaning that a single biopsy may not accurately represent the overall tumour immune microenvironment (47).

Tumour mutational burden has emerged as a complementary biomarker, with higher TMB levels correlating with improved ICI responses in several malignancies, including NSCLC, melanoma, and urothelial carcinoma (30). The biological rationale is that tumours with higher mutational loads generate more neoantigens, thereby increasing the likelihood of immune recognition. However, the clinical utility of TMB is limited by several factors. First, there is no universally accepted threshold for defining “high” TMB, with cut-offs ranging from 10 to 20 mutations per megabase across different studies and tumour types (31). Second, TMB assessment varies depending on the sequencing platform used, with whole-exome sequencing and targeted gene panels yielding different results (48). Third, TMB has demonstrated inconsistent predictive performance across clinical trials; for example, the KEYNOTE-158 trial demonstrated a correlation between high TMB and pembrolizumab response across multiple tumour types, whereas other studies have failed to replicate this association in specific histologies (49). Clinicians must therefore navigate considerable uncertainty when using these biomarkers for treatment decisions, and neither PD-L1 expression nor TMB should be considered absolute predictors of response or resistance.

Emerging biomarkers offer promise for improving patient selection but require further validation. Immune gene expression signatures, such as the tumour inflammation signature (TIS) and interferon-gamma-related gene panels, assess the inflammatory status of the tumour microenvironment and have shown predictive value independent of PD-L1 expression in several tumour types (50). Circulating tumour DNA (ctDNA) represents a minimally invasive approach to monitoring treatment response and detecting resistance; early ctDNA dynamics during ICI therapy have been associated with clinical outcomes, and ctDNA clearance may serve as an early indicator of durable response (51). The gut microbiome has also emerged as a potential modulator of ICI efficacy, with specific bacterial taxa such as Akkermansia muciniphila and Faecalibacterium prausnitzii associated with favourable responses to PD-1 blockade (52). However, microbiome research in this context remains in its early stages, with significant variability in study methodologies, patient populations, and analytical approaches limiting the generalizability of findings (53). Other investigational biomarkers include the neutrophil-to-lymphocyte ratio, lactate dehydrogenase levels, and multiplex immunohistochemistry panels assessing tumour-infiltrating lymphocyte density and composition (32, 54). The integration of multiple biomarkers into composite predictive models, potentially enhanced by artificial intelligence and machine learning algorithms, represents a promising direction for achieving more accurate and individualized treatment selection (15, 34).

### Combination therapy evidence from included studies

Four of the 15 included studies directly evaluated combination nivolumab plus ipilimumab, providing the strongest evidence base for dual checkpoint blockade within this review. In advanced melanoma, Wolchok et al. (14) reported 3-year OS of 58% with combination therapy (14), Larkin et al. (21) demonstrated OS of 11.5 months versus 6.9 months with monotherapy (21), and Postow et al. (1) showed 12-month OS of 74% versus 53% with ipilimumab alone (1). In renal cell carcinoma, Motzer et al. (55) reported 18-month OS of 75% versus 60% with sunitinib (55). These four studies consistently demonstrated that combination therapy achieved superior survival outcomes compared with monotherapy or standard comparators.

However, all four combination therapy studies also reported substantially higher toxicity rates compared with monotherapy arms. Wolchok et al. (14) documented severe irAEs (14), Larkin et al. (21) reported fatigue and colitis (21), Postow et al. (1) observed colitis and pneumonitis (1), and Motzer et al. (55) noted diarrhea and hepatitis (55). This consistent pattern across the included studies highlights the efficacy-toxicity trade-off inherent to dual checkpoint blockade.

Notably, none of the 15 included studies evaluated ICI combined with chemotherapy, ICI combined with targeted therapy, or novel checkpoint targets such as LAG-3, TIM-3, or TIGIT. The combination therapy evidence within this review is therefore limited to dual PD-1/CTLA-4 blockade in melanoma and renal cell carcinoma.

#### Contextualization with broader literature and future directions

Beyond the included studies, the combination of ICIs with chemotherapy has demonstrated efficacy in NSCLC, where pembrolizumab combined with platinum-based chemotherapy resulted in improved OS compared to chemotherapy alone (32). Novel immune checkpoints such as LAG-3, TIM-3, and TIGIT are under investigation with the goal of overcoming resistance mechanisms and expanding the scope of immunotherapy (33). However, early-phase trial results have yielded mixed outcomes, and the added benefit over existing agents requires validation in larger, well-designed studies (33, 52).

Emerging strategies incorporating artificial intelligence and machine learning aim to improve patient selection accuracy and optimize treatment outcomes (15). Artificial intelligence-based integration of radiomics, genomics, and clinical data may enable identification of response and toxicity biomarkers, facilitating personalized immunotherapy (34).

While combination strategies offer the potential for enhanced efficacy, they also introduce greater complexity in terms of toxicity management and cost considerations. The increased incidence of high-grade irAEs observed with dual checkpoint blockade necessitates careful patient selection and robust infrastructure for toxicity monitoring and management (21, 50). Furthermore, the optimal sequencing of combination therapies, the identification of patients most likely to benefit from intensified regimens versus those who may achieve equivalent outcomes with monotherapy, and the cost-effectiveness of these approaches remain areas of active investigation (51). As the therapeutic landscape becomes increasingly complex, there is a pressing need for head-to-head comparative trials and real-world effectiveness studies to guide evidence-based treatment selection (53).

### Clinical implications

The findings from the 15 included studies have several important implications for clinical practice.

First, the heterogeneity in efficacy outcomes observed across the six tumour types represented in this review — with OS improvements ranging from 2.4 months in head and neck SCC (17) to over 5 months in renal cell carcinoma (6) — underscores the necessity for individualized treatment selection based on tumour histology, molecular characteristics, and patient-specific factors rather than applying a uniform approach to ICI therapy.

Second, the consistent reporting of irAEs across all 15 included studies, with higher rates observed in the four combination therapy trials (1, 14, 21, 55), highlights the importance of comprehensive patient education, proactive monitoring, and low thresholds for initiating diagnostic workup when symptoms suggestive of irAEs arise. Real-world evidence suggests that irAE rates in routine clinical practice may exceed those reported in controlled trials (10, 41).

Third, the NSCLC studies included in this review demonstrated that PD-L1 expression was predictive of pembrolizumab efficacy (5, 16, 20), whereas the melanoma and renal cell carcinoma studies showed benefit regardless of PD-L1 status (4, 14, 21, 55). Clinicians should therefore interpret biomarker results within the broader clinical context and tumour-specific evidence rather than relying on them as absolute predictors of response. Notably, the utility of PD-L1 and TMB as predictors of immune-related adverse events is not established, and these biomarkers should not currently be used for toxicity risk stratification outside of investigational settings.

Fourth, the increased toxicity associated with combination nivolumab plus ipilimumab in the four included studies (1, 6, 14, 21) necessitates careful patient selection, with consideration of performance status, comorbidities, and patient preferences when weighing the potential benefits against risks.

Finally, given the multi-organ nature of irAEs documented across the included studies — including colitis, pneumonitis, hepatitis, and endocrinopathies — the establishment of multidisciplinary teams involving oncologists, organ specialists, and supportive care providers is essential for optimal irAE management and should be considered a standard component of ICI treatment programs (10, 28, 43).

## Limitations of this review

Several limitations of this systematic review should be acknowledged. First, despite comprehensive search strategies across five databases, publication bias may have influenced the available evidence, as positive trials are more likely to be published than negative studies (54). Second, the 15 included studies demonstrated substantial heterogeneity in study designs (13 RCTs, 2 cohort studies), patient populations, tumor types, comparator arms, and outcome definitions, which precluded formal meta-analysis and limited direct cross study comparisons. Third, the included studies were concentrated in melanoma (5 studies), NSCLC (4 studies), and renal cell carcinoma (2 studies), with limited representation of other malignancies, restricting the generalizability of findings. Fourth, the search timeframe (January 2010 to October 2024) means that some recently published data including long-term follow up from landmark trials and studies evaluating novel checkpoint targets, may not have been captured. Fifth, none of the 15 included studies prospectively evaluated tumor mutational burden as a selection biomarker, limiting conclusions regarding this emerging predictive marker. Sixth, the certainty of evidence varied across outcomes: while overall survival data in melanoma and NSCLC were supported by moderate-certainty evidence from multiple RCTs, conclusions regarding safety outcomes and biomarker-toxicity associations were based on low-certainty evidence due to inconsistent irAE definitions, heterogeneous reporting, and limited prospective data, and this was reflected in the strength of our conclusions. Seventh, the exclusion of non- English publications may have resulted in the omission of relevant studies from non-English-speaking regions.

## Conclusion

This systematic review of 15 studies (13 RCTs, 2 cohort studies) demonstrates that immune checkpoint inhibitors provide clinically meaningful survival benefits across multiple tumor types, including melanoma, NSCLC, renal cell carcinoma, head and neck SCC, urothelial carcinoma, and Hodgkin lymphoma. Overall survival improvements ranged from 2.4 months to over 5 months compared with standard therapies, with combination nivolumab plus ipilimumab achieving the highest response rates but also the greatest toxicity burden.

All 15 included studies reported immune-related adverse events, with combination therapy consistently associated with higher rates of severe toxicities including colitis, pneumonitis, and hepatitis. The NSCLC studies demonstrated that PD-L1 expression was predictive of pembrolizumab efficacy, whereas melanoma and renal cell carcinoma studies showed benefit regardless of PD-L1 status, highlighting the tumor-specific nature of biomarker utility.

The heterogeneity of the included evidence, limitations in current biomarker strategies, and gaps in toxicity management guidelines underscore the need for continued research efforts. While PD-L1 expression and TMB are established biomarkers for predicting ICI response, their role in predicting immune-related adverse events remains investigational and is not supported by robust evidence from the included studies or the broader literature. Future research should focus on optimizing combination ICI regimens, developing validated composite biomarkers integrating PD-L1, TMB, and emerging markers for response prediction, identifying distinct biomarkers for toxicity risk stratification, and establishing evidence-based irAE management protocols through prospective studies.

## Data Availability

The raw data supporting the conclusions of this article will be made available by the authors, without undue reservation.
