# Supplementary material for: Immune Checkpoint Inhibitors: efficacy, safety, and biomarkers - a systematic review
Source: Front Oncol. 2026 May 20;16:1789760. doi: 10.3389/fonc.2026.1789760 (PMC13229778; doi:10.3389/fonc.2026.1789760)
Supplement: Supplementary file 1 [file Table1.docx]

**Supplementary Table S1: Complete Search Strategies for All Databases**

**Search date:** October 2024

**Total records identified:** 4,216

**S1a. PubMed/MEDLINE**

**Records retrieved:** 1,180

**Interface:** PubMed (National Library of Medicine)

| **Line** | **Search Terms** |
| --- | --- |
| #1 | "Immune Checkpoint Inhibitors"[MeSH Terms] OR "immune checkpoint inhibitor*"[tiab] |
| #2 | "Programmed Cell Death 1 Receptor"[MeSH Terms] OR "PD-1"[tiab] OR "PD1"[tiab] OR "anti-PD-1"[tiab] |
| #3 | "B7-H1 Antigen"[MeSH Terms] OR "PD-L1"[tiab] OR "PDL1"[tiab] OR "anti-PD-L1"[tiab] |
| #4 | "CTLA-4 Antigen"[MeSH Terms] OR "CTLA-4"[tiab] OR "CTLA4"[tiab] |
| #5 | "Ipilimumab"[MeSH Terms] OR ipilimumab[tiab] OR Yervoy[tiab] |
| #6 | "Nivolumab"[MeSH Terms] OR nivolumab[tiab] OR Opdivo[tiab] |
| #7 | pembrolizumab[tiab] OR Keytruda[tiab] OR "MK-3475"[tiab] |
| #8 | atezolizumab[tiab] OR Tecentriq[tiab] OR "MPDL3280A"[tiab] |
| #9 | durvalumab[tiab] OR Imfinzi[tiab] OR "MEDI-4736"[tiab] |
| #10 | avelumab[tiab] OR Bavencio[tiab] |
| #11 | cemiplimab[tiab] OR Libtayo[tiab] |
| #12 | dostarlimab[tiab] OR Jemperli[tiab] |
| #13 | toripalimab[tiab] OR tislelizumab[tiab] |
| #14 | #1 OR #2 OR #3 OR #4 OR #5 OR #6 OR #7 OR #8 OR #9 OR #10 OR #11 OR #12 OR #13 |
| #15 | "Neoplasms"[MeSH Terms] OR cancer[tiab] OR carcinoma[tiab] OR tumor[tiab] OR tumour[tiab] OR malignan*[tiab] |
| #16 | #14 AND #15 |
| #17 | "randomized controlled trial"[pt] OR "controlled clinical trial"[pt] OR randomized[tiab] OR placebo[tiab] OR randomly[tiab] OR trial[ti] OR cohort[tiab] |
| #18 | animals[MeSH Terms] NOT humans[MeSH Terms] |
| #19 | #17 NOT #18 |
| #20 | #16 AND #19 |
| #21 | #20 AND ("2010/01/01"[Date - Publication] : "2024/10/31"[Date - Publication]) |
| #22 | #21 AND English[Language] |

**S1b. Embase (via OvidSP)**

**Records retrieved:** 1,350

**Interface:** OvidSP

| **Line** | **Search Terms** |
| --- | --- |
| #1 | 'immune checkpoint inhibitor'/exp OR 'immune checkpoint inhibitor*':ti,ab |
| #2 | 'programmed cell death 1 receptor'/exp OR 'pd-1':ti,ab OR 'pd1':ti,ab |
| #3 | 'programmed cell death ligand 1'/exp OR 'pd-l1':ti,ab OR 'pdl1':ti,ab |
| #4 | 'cytotoxic t lymphocyte antigen 4'/exp OR 'ctla-4':ti,ab OR 'ctla4':ti,ab |
| #5 | 'ipilimumab'/exp OR 'nivolumab'/exp OR 'pembrolizumab'/exp OR 'atezolizumab'/exp OR 'durvalumab'/exp OR 'avelumab'/exp OR 'cemiplimab'/exp |
| #6 | #1 OR #2 OR #3 OR #4 OR #5 |
| #7 | 'neoplasm'/exp OR cancer:ti,ab OR carcinoma:ti,ab OR tumor:ti,ab OR malignan*:ti,ab |
| #8 | #6 AND #7 |
| #9 | 'randomized controlled trial'/exp OR 'cohort analysis'/exp OR random*:ti,ab OR placebo:ti,ab |
| #10 | #8 AND #9 |
| #11 | #10 AND/py AND [english]/lim |
| #12 | #11 NOT ('conference abstract'/it OR 'letter'/it OR 'editorial'/it) |

**S1c. Scopus**

**Records retrieved**: 820

**Interface**: Scopus (Elsevier)

**Search string:** TITLE-ABS-KEY(("immune checkpoint inhibitor<em>" OR "PD-1" OR "PD-L1" OR "CTLA-4" OR ipilimumab OR nivolumab OR pembrolizumab OR atezolizumab OR durvalumab) AND (cancer OR carcinoma OR tumor OR neoplasm OR malignancy) AND (randomized OR "clinical trial" OR cohort)) AND PUBYEAR > 2009 AND PUBYEAR < 2025 AND LANGUAGE(english)</em>

**S1d. Web of Science Core Collection**

**Records retrieved:** 650

**Interface:** Web of Science (Clarivate)

**Search string:** TS=(("immune checkpoint inhibitor<em>" OR "PD-1" OR "PD-L1" OR "CTLA-4" OR ipilimumab OR nivolumab OR pembrolizumab OR atezolizumab OR durvalumab) AND (cancer OR carcinoma OR tumor OR neoplasm OR malignancy) AND (randomized OR "clinical trial" OR cohort))</em>

**Filters applied:**

- Publication Years: 2010–2024
- Languages: English
- Document Types: Article

**S1e. Cochrane Central Register of Controlled Trials (CENTRAL)**

**Records retrieved:** 216

**Interface:** Cochrane Library (Wiley)

| **Line** | **Search Terms** |
| --- | --- |
| #1 | MeSH descriptor: [Immune Checkpoint Inhibitors] explode all trees |
| #2 | ("immune checkpoint inhibitor*"):ti,ab,kw |
| #3 | ("PD-1" OR "PD-L1" OR "CTLA-4"):ti,ab,kw |
| #4 | (ipilimumab OR nivolumab OR pembrolizumab OR atezolizumab OR durvalumab):ti,ab,kw |
| #5 | #1 OR #2 OR #3 OR #4 |
| #6 | MeSH descriptor: [Neoplasms] explode all trees |
| #7 | (cancer OR carcinoma OR tumor OR malignancy):ti,ab,kw |
| #8 | #6 OR #7 |
| #9 | #5 AND #8 |
| #10 | Publication Year: 2010–2024 |

**S1f. ClinicalTrials.gov**

**Interface:** ClinicalTrials.gov (U.S. National Library of Medicine)

**Search terms:** immune checkpoint inhibitor OR PD-1 OR PD-L1 OR CTLA-4 OR ipilimumab OR nivolumab OR pembrolizumab OR atezolizumab OR durvalumab

**Filters applied:**

- Study type: Interventional
- Status: Completed
- Results: With results
- First posted: 01/01/2010 to 10/31/2024

## Summary of Records by Database

| **Database** | **Records Retrieved** |
| --- | --- |
| PubMed/MEDLINE | 1,180 |
| Embase | 1,350 |
| Scopus | 820 |
| Web of Science | 650 |
| Cochrane CENTRAL | 216 |
| **Total** | **4,216** |

**Supplementary Table SX: Risk of Bias Assessment for Included Studies**

**Randomized Controlled Trials (n=13) — Assessed using Cochrane Risk of Bias 2 (RoB 2) Tool**

RoB 2 Domains:

- D1: Randomization process
- D2: Deviations from intended interventions
- D3: Missing outcome data
- D4: Measurement of the outcome
- D5: Selection of the reported result

| **Study** | **Design** | **D1: Randomization** | **D2: Deviations** | **D3: Missing Data** | **D4: Outcome Measurement** | **D5: Selective Reporting** | **Overall Judgment** |
| --- | --- | --- | --- | --- | --- | --- | --- |
| Hodi et al. 2010 (4) | RCT | Low | Low | Low | Some concerns | Low | Some concerns |
| Garon et al. 2015 (5) | RCT | Low | Low | Low | Low | Low | Low risk |
| Motzer et al. 2015 (6) | RCT | Low | Low | Low | Low | Low | Low risk |
| Wolchok et al. 2017 (14) | RCT | Low | Low | Low | Low | Low | Low risk |
| Reck et al. 2016 (16) | RCT | Low | Low | Low | Low | Low | Low risk |
| Ferris et al. 2016 (17) | RCT | Low | Low | Low | Low | Low | Low risk |
| Bellmunt et al. 2017 (18) | RCT | Low | Low | Low | Low | Low | Low risk |
| Herbst et al. 2016 (20) | RCT | Low | Low | Low | Low | Low | Low risk |
| Larkin et al. 2015 (21) | RCT | Low | Low | Low | Low | Low | Low risk |
| Borghaei et al. 2015 (22) | RCT | Low | Low | Low | Low | Low | Low risk |
| Eggermont et al. 2018 (23) | RCT | Low | Low | Low | Low | Low | Low risk |
| Motzer et al. 2018 (55) | RCT | Low | Low | Low | Low | Low | Low risk |
| Postow et al. 2015 (1) | RCT | Low | Low | Low | Low | Low | Low risk |

**Cohort Studies (n=2) — Assessed using Newcastle-Ottawa Scale (NOS)**

NOS Domains:

- Selection (maximum 4 stars)
- Comparability (maximum 2 stars)
- Outcome (maximum 3 stars)
- Total score: 0–3 = Low quality; 4–6 = Moderate quality; 7–9 = High quality

| **Study** | **Design** | **Selection (★★★★)** | **Comparability (★★)** | **Outcome (★★★)** | **Total Score** | **Quality Rating** |
| --- | --- | --- | --- | --- | --- | --- |
| Ansell et al. 2015 (7) | Cohort | ★★★★ | ★ | ★★★ | 8/9 | High |
| Ribas et al. 2016 (19) | Cohort | ★★★★ | ★ | ★★★ | 8/9 | High |

**Notes:**

- Hodi et al. 2010 was rated "Some concerns" overall due to open-label design potentially affecting outcome measurement (D4), as progression-free survival was investigator-assessed rather than by independent central review.
- Both cohort studies received high-quality ratings (8/9 stars) but were downgraded one star in comparability due to limited adjustment for potential confounders.
- All other RCTs were rated as low risk of bias across all domains.

**Supplementary Table SY: GRADE Summary of Findings**

**Population:** Patients with advanced malignancies receiving immune checkpoint inhibitors

**Intervention:** PD-1, PD-L1, or CTLA-4 inhibitors (alone or in combination)

**Comparison:** Standard chemotherapy, targeted therapy, or placebo

| **Outcome** | **No. of Studies** | **Total Participants** | **Effect Estimate** | **Certainty of Evidence** | **Reasons for Downgrading** |
| --- | --- | --- | --- | --- | --- |
| **Overall Survival (Melanoma)** | 5 | 3,566 | OS range: 10.1–11.5 months (monotherapy); 58% 3-year OS (combination); HR favoring ICI in all studies | ⊕⊕⊕◯ **Moderate** | Downgraded one level for some concerns regarding risk of bias (open-label designs with investigator-assessed endpoints in some studies) |
| **Overall Survival (NSCLC)** | 4 | 2,226 | OS range: 12.2–13.7 months vs 8.2–10.3 months (chemotherapy); HR 0.59–0.71 favoring ICI | ⊕⊕⊕◯ **Moderate** | Downgraded one level for inconsistency (variable magnitude of benefit across PD-L1 expression subgroups) |
| **Overall Survival (RCC)** | 2 | 1,917 | OS: 25 months vs 19.6 months (nivolumab vs everolimus); 75% vs 60% 18-month OS (combination vs sunitinib) | ⊕⊕⊕◯ **Moderate** | Downgraded one level for imprecision (only 2 studies, though both large RCTs) |
| **Overall Survival (Other tumors)** | 4 | 1,093 | OS improvements: 2.4 months (HNSCC); 2.9 months (urothelial); ORR 87% (Hodgkin); DFS 75.4% vs 61% (adjuvant melanoma) | ⊕⊕◯◯ **Low** | Downgraded two levels for indirectness (single study per tumor type) and imprecision |
| **Objective Response Rate (All tumors)** | 15 | 8,802 | ORR range: 19–87% depending on tumor type and regimen; highest in Hodgkin lymphoma (87%), lowest in solid tumors with monotherapy (19–34%) | ⊕⊕◯◯ **Low** | Downgraded two levels for inconsistency (substantial heterogeneity across tumor types and regimens) and indirectness (variable definitions of response) |
| **Immune-Related Adverse Events (Grade 3–4)** | 15 | 8,802 | Incidence range: 10–59%; highest with combination nivolumab + ipilimumab (46–59%); lowest with PD-1 monotherapy (10–15%) | ⊕⊕◯◯ **Low** | Downgraded two levels for inconsistency (variable irAE definitions and CTCAE versions across studies) and imprecision (wide confidence intervals for rare events) |
| **Biomarker-Toxicity Association (PD-L1/TMB as predictors of irAEs)** | 0 directly; contextual evidence only | — | No included study prospectively evaluated PD-L1 or TMB as predictors of irAE risk; external meta-regression found no significant association between TMB and toxicity (56) | ⊕◯◯◯ **Very Low** | Downgraded four levels for indirectness (no direct evidence from included studies), imprecision (limited data), risk of bias (retrospective analyses only), and publication bias (positive predictive studies more likely published) |

**Footnotes:**

- GRADE certainty ratings: ⊕⊕⊕⊕ High; ⊕⊕⊕◯ Moderate; ⊕⊕◯◯ Low; ⊕◯◯◯ Very Low
- HR = hazard ratio; OS = overall survival; ORR = objective response rate; DFS = disease-free survival; RCC = renal cell carcinoma; HNSCC = head and neck squamous cell carcinoma; irAE = immune-related adverse event; CTCAE = Common Terminology Criteria for Adverse Events
- Publication bias was assessed qualitatively; formal funnel plot analysis was not possible due to the absence of meta-analysis
- The "Biomarker-Toxicity Association" outcome was rated very low certainty because no included study directly evaluated this question; the conclusion is based on external contextual evidence (reference 56)

**Table 3: Risk of Bias Assessment for Included Studies**

**Panel A: Randomized Controlled Trials (Cochrane RoB 2 Tool)**

| **Study** | **D1: Randomization Process** | **D2: Deviations from Interventions** | **D3: Missing Outcome Data** | **D4: Measurement of Outcome** | **D5: Selection of Reported Result** | **Overall Risk of Bias** |
| --- | --- | --- | --- | --- | --- | --- |
| Hodi et al. 2010 (4) | Low | Low | Low | Some concerns | Low | Some concerns |
| Garon et al. 2015 (5) | Low | Low | Low | Low | Low | Low |
| Motzer et al. 2015 (6) | Low | Low | Low | Low | Low | Low |
| Wolchok et al. 2017 (14) | Low | Low | Low | Low | Low | Low |
| Reck et al. 2016 (16) | Low | Low | Low | Low | Low | Low |
| Ferris et al. 2016 (17) | Low | Low | Low | Low | Low | Low |
| Bellmunt et al. 2017 (18) | Low | Low | Low | Low | Low | Low |
| Herbst et al. 2016 (20) | Low | Low | Low | Low | Low | Low |
| Larkin et al. 2015 (21) | Low | Low | Low | Low | Low | Low |
| Borghaei et al. 2015 (22) | Low | Low | Low | Low | Low | Low |
| Eggermont et al. 2018 (23) | Low | Low | Low | Low | Low | Low |
| Motzer et al. 2018 (55) | Low | Low | Low | Low | Low | Low |
| Postow et al. 2015 (1) | Low | Low | Low | Low | Low | Low |

**Panel B: Cohort Studies (Newcastle-Ottawa Scale)**

| **Study** | **Selection (max 4★)** | **Comparability (max 2★)** | **Outcome (max 3★)** | **Total Score** | **Quality** |
| --- | --- | --- | --- | --- | --- |
| Ansell et al. 2015 (7) | ★★★★ | ★ | ★★★ | 8/9 | High |
| Ribas et al. 2016 (19) | ★★★★ | ★ | ★★★ | 8/9 | High |
